# A Clinical Study of Purulent Ophthalmia

**Published:** 1887-11

**Authors:** John T. Oldham

**Affiliations:** Louisville, Ky.


### A CLINICAL STUDY OF PURULENT OPHTHALMIA.

BY JOHN T. OLDHAM, M. D., LOUISVILLE, KY.

Having studied a large number of samples of pus in suppurative conjunctivitis, I became interested a year ago in some experiments designed to test the value of different methods of treatment of conjunctivitis. These experiments were conducted *pari passu* by artificial cultures of the micro-organisms in conjunctival pus and the course of the inflammation in the eyes of ten persons, seven of them inmates of an orphan asylum and three in private practice, all of them occurring about the same time. The cases were isolated and the treatment fully carried out.

C. C., forty-eight hours old. Called with Dr. Dudley S. Reynolds on the 9th cf October, 1886. A quantity of pus was taken from the right eye, which was the one worst affected, the lids being swollen, the conjunctiva chemosed, and an abundant flow of thick yellow pus from between the lids of both eyes. Sodium borate, five grains ; pure carbolic acid, one drop ; distilled water, one ounce, were ordered to be instilled every fifteen minutes between the lids. An ointment of fifteen grains of boric acid to half an ounce of petrolatum, was directed to be smeared upon the eyelash after each application of the collyrium. Twenty-four hours later the swelling had almost entirely disappeared from the lids, there was but little discharge of pus, and the patient was greatly improved. Forty hours elapsed, and there was no swelling, no discharge of pus, the eyes opened naturally, and the patient was discharged convalescent. Samples of the matter were rich in the gonococous of Neisser.

Half an hour after my first visit to this patient, a man came into the office with a grain of emery embedded in the eornea. I instilled a solution of cocaine and removed the emery. Next morning he came with suppuration of the cornea. The collyrium of borax and carbolic acid was ordered to be used every hour. He made no improvement. The next day the point of suppuration in the cornea was touched with the end of a silver probe previously dipped in pure carbolic acid. Twenty-four hours later the patient was convalescent.

M. V., mulatto, eight years of age, brought to the Hospital College clinic with purulent conjunctivitis in the right eye. The collyrium of borax and carbolic acid being ordered every hour, the patient was directed to report the next day; but failing to do so, I visited her in the afternoon, and found the other eye affected. I prescribed one grain of corrosive sublimate, six grains of ammonia muriate, and eight ounces of distilled water. With this I cleansed the conjunctival sac of both eyes thoroughly and directed the operation to be repeated every half hour. In two days this patient was convalescent. Four days afterward reinoculation occurred and a violent relapse, more severe than the first attack. Continued use of the bichloride solution brought on complete recovery.

J. H., a young man twenty-two years of age, had gonorrhœa for the past six weeks, when suddenly the left eye became inflamed.

October 29 he appeared at the office with the left eye closed from swollen lids and a profuse discharge of pus. The lower half of cornea was gray, and it was considered in imminent danger of sloughing. The mercury bichloride solution, one grain to four ounces of water, with sufficient amount of ammonia muriate to make a solution, was ordered to be instilled every hour. He was directed to employ a nurse to sit up all night with him and to continue the treatment. In five days he was convalescent.

The cases at the orphan asylum were treated in a precisely similar manner, each using the corrosive sublimate solution, varying in strength from one grain to eight ounces of water, to a grain in twelve ounces, the frequency of the application and the strength of the solution being proportioned to the severity of the attack. I know of two cases in which ulceration of the cornea had already commenced, yet in no case did it fail to yield to the mercury bichloride solution except where a strongly marked circumiscribed suppuration existed, and then pure carbolic acid was applied by dipping a silver probe into the acid and pressing it to the bottom of the ulcer.

These observations, though not entirely new, are in some respects unusual. I present them with the view of suggesting the importance of relying upon the remedies named rather than severe caustic applications in the treatment of the different forms of purulent conjunctivitis with, and without corneal applicat ons.—*Medical Standard.*

